# The effect of mobile personalised texting versus non-personalised texting on the caries risk of underprivileged adults: a randomised control trial

**DOI:** 10.1186/s12903-019-0729-1

**Published:** 2019-03-12

**Authors:** Makiko Nishi, Virginia Kelleher, Michael Cronin, Finbarr Allen

**Affiliations:** 1 Non-profit Organisation “Promoting Scientific Assessment in Prevention of Tooth Decay and Gum Disease”, Tokyo, Japan; 2 Oral Health Services Research Centre, University College Cork, Cork, Republic of Ireland; 3 School of Mathematical Sciences, University College Cork, Cork, Republic of Ireland; 4 Prosthodontics and Oral Rehabilitation, Cork Dental School and Hospital, University College Cork, Cork, Republic of Ireland

**Keywords:** Dental caries, Risk factors, Vulnerable populations, Dental health education, Risk reduction behaviour, Ireland, Adult, Cariogram

## Abstract

**Background:**

In the Republic of Ireland (RoI), fluoridation has been effective and efficient for caries prevention at population level, regardless of income status; however, at individual level it still has limitations. This study aimed to compare personalised versus non-personalised text messaging on ‘*chance of avoiding new cavities*’ with the Cariogram, a computer-based caries risk assessment (CRA) model, in an economically disadvantaged adult population in the RoI.

**Methods:**

The intervention was via a CRA summary letter plus 24 weekly personalised mobile-phone short text messages (text messages) based on the individual’s CRA, compared with a non-personalised approach via a non-personalised letter and a predetermined, fixed set of 24 weekly text messages. The study was designed as a two-arm parallel-group, single-blinded (assessor), randomised controlled study in County Cork, RoI. The primary outcome was a comparison of ‘*chance of avoiding new cavities*’ calculated by the Cariogram with clinical examination, interview, CRT® (Ivoclar Vivadent, Liechtenstein) and three-day food diary between the two groups at follow-up. We combined stratified randomisation with blocked randomisation for 171 participants who completed baseline. Of them, 111 completed follow-up and were analysed (56 and 55 from the personalised and non-personalised groups, respectively). Due to protocol violations, both intent-to-treat (ITT) and per-protocol analyses were conducted.

**Results:**

The ITT analysis did not show a personalised intervention effect on ‘*chance of avoiding new cavities*’. Of the secondary outcome measures, only the stimulated saliva flow factor showed a personalised intervention effect, *p* = 0.036, OR = 0.3 (95% CI = 0.1, 0.9). The per-protocol analysis with 21 personalised and 33 non-personalised participants within two-message deviations showed no significant effect on ‘*chance of avoiding new cavities*’.

**Conclusions:**

The null hypothesis in regard to the primary outcome for both ITT and per-protocol analyses was not rejected; however, as the minimal clinically important difference was included in the 95% CI for the per-protocol analysis, replication studies will be worth conducting to explore the potential of mobile devices for individual caries risk reduction.

**Trial registration:**

University Hospital Medical Information Network Clinical Trials Registry (UMIN000027253) on 10 May 2017. The study was retrospectively registered.

**Electronic supplementary material:**

The online version of this article (10.1186/s12903-019-0729-1) contains supplementary material, which is available to authorized users.

## Background

The incidence of dental caries, a preventable disease [1], is strongly associated with social and economic deprivation [2]. In the Republic of Ireland (RoI), fluoridated water has been effective and efficient for caries prevention at population level, regardless of income status [3]. Although the most recent data on 15-year-olds in the RoI was collected 17 years ago, it remained a concern that by age 15 approximately three quarters of adolescents with fluoridated water supplies in the RoI have experienced dental caries [4]. To compensate for this limitation of water fluoridation, caries prevention based on an individual’s caries risk assessment (CRA) could be of value to the individual [5].

Even within the lower socioeconomic groups, there are multiple caries risk factors which may vary from person to person and may change during a person’s lifetime; therefore, it seems reasonable that applying a personalised preventive approach could be effective [6]. Mobile health (mHealth) has enormous potential for conducting personalised approaches to disease prevention and management [7]. Mobile devices allow low cost interventions and are a means of providing individual level support to health care consumers in order to increase healthy behaviour [8]. An automated system can send bulk personalised text messages using an algorithm based on patients’ information to patients anywhere and anytime. Personalised messages exhibited the largest effect size in a meta-analysis on efficacy of text messages for health promotion [9].

In dentistry, for example, mobile-phone text messaging improved tooth brushing frequencies among unemployed young adults [10], oral health knowledge and behaviour in mothers of young children [11] and plaque removal in orthodontic patients [12]. To the best of our knowledge, no study has previously been conducted on mHealth interventions for a personalised approach based on CRA.

The Cariogram, a validated computer-based CRA model [6], “*is a graphical picture illustrating in an interactive way the individual’s/patient’s risk for developing new caries in the future, simultaneously expressing to what extent different etiological factors of caries affect the caries risk for that particular patient.”* [13]. The Cariogram calculates the four risk-sector values – ‘Diet’, ‘Bacteria’, ‘Susceptibility’ and ‘Circumstances’, based on combinations of nine risk parameters as follows: ‘Diet’ is based on ‘Diet contents’ and ‘Diet frequency’; ‘Bacteria’ is based on ‘Plaque amount’ and ‘Mutans streptococci’; ‘Susceptibility’ is based on ‘Fluoride programme’, ‘Saliva secretion’ and ‘Saliva buffer capacity’; ‘Circumstances’ is based on ‘Caries experience’ and ‘Related diseases’. These nine parameters plus the ‘Clinical judgement’ parameter are scored 0, 1, 2 or 0, 1, 2, 3, and given different weights; the scores are not simply added together. The total of the four risk-sector values subtracted from 100 equals ‘*chance of avoiding new cavities*’.

The aim of this study was to compare personalised versus non-personalised text messaging on ‘*chance of avoiding new cavities*’ with the Cariogram, in an economically disadvantaged adult population. The null hypothesis to be tested is that no difference would exist in ‘*chance of avoiding new cavities*’ between the group receiving personalised information and a comparison group receiving non-personalised information, among economically disadvantaged adults.

## Methods

### Study design

The study design was a two-arm parallel-group, single-blinded (assessor), randomised controlled study with a 1:1 allocation ratio comparing personalised (test) and non-personalised (control) caries preventive advice. Ethical approval was granted by the Clinical Research Ethics Committee of the Cork Teaching Hospitals of University College Cork (UCC) (ECM 4 (r) 12/08/14). The study was conducted in compliance with the principles of the Declaration of Helsinki. The study protocol is available on the website of the Oral Health Services Research Centre (OHSRC) [14]. All files including personal information were coded. Detailed calibration examination and baseline procedures, subject characteristics, and data collection have already been described elsewhere [15, 16]. Protocol violations by the computer programmer occurred after trial commencement; an additional file shows the protocol violations in more detail [see Additional file 1].

### Subjects

An a priori sample size calculation was performed after systematically searching literature through PubMed on studies using the Cariogram among adults. Based on two previous studies [17, 18], we set a significance level of 5% (two-sided), a power for that detection of 80%, a control response of 36 (‘*chance of avoiding new cavities*’), a standard deviation of 21.6 and a change-relative-to-control mean of 30% for the two-sample *t* test. We considered Δ11 (= 36*30%) of ‘*chance of avoiding new cavities*’ as the minimal clinically important difference (MCID). The required size for the study was 128 subjects randomised into two groups of 64 subjects. As it was expected we would recruit even numbers of participants from each dental practitioner, clustering by dental practitioners was not considered for the sample calculation.

The pre-determined inclusion criteria for patient participants were (1) those who were ready to give consent, (2) 19–70 years of age, (3) medical-card holder (i.e. proxy for economically disadvantaged status; a medical-card holder is entitled to a range of health services free of charge in the RoI), (4) at least 20 teeth present, (5) not pregnant and (6) ability to use text messages. The eight trained and calibrated dental practitioners (Dentists A to H) in County Cork, RoI, who were volunteers with an interest in practice-based research, recruited medical-card-holder patients and obtained written informed consent from each patient participant. The Kappa statistics for inter-examiner reliability ranged from 0.91–1.00 and 0.54–0.94 for tooth status and coronal surface caries condition, respectively. For root caries, the Kappa statistics for inter-examiner reliability were 0.37–0.48. Allowing for a non-response rate of 33%, 191 participants (62 men and 129 women) were recruited.

We combined stratified randomisation with blocked randomisation. The block size was randomly varying. After consultation with the statistician who had looked at the first group of participants’ data (*n* = 52) before randomisation commenced, we decided to stratify the participants into five groups (‘*chance of avoiding new cavities*’ of 0–20, 21–40, 41–50, 51–60, 61–100). The statistician generated random numbers for stratified and blocked randomisation using Proc Surveyselect, SAS, Version 9.4 (SAS Institute Inc., Cary, NC). Details on the allocation concealment is presented in Additional file 2.

### Interventions

The text messages covered the four caries risk-sectors in accordance with the Cariogram output. We created more than 96 (= 24 weeks * 4 risk-sectors) educational text messages, and assigned a priority ranking to each message. Each message was kept within the maximum of 160 characters. The draft messages were prepared by one dentist and were based on available evidence from literature [19, 20], public websites [21–28], the Cariogram Manual [13], and educational emails of a non-profitable organisation [29] and Rapport Builder® (Oral Care Inc., Japan) [30]. The text messages were checked and revised by one editor, one psychologist, two neuroscientists and two dentists, then piloted with three staff members in the OHSRC and one dental student. Following a trial-sending of the actual text messages to three dental students and one occupational therapist, the text messages were finalised on 26 November 2014. Examples of the text messages are presented in Additional file 3.

Using the Cariogram output at baseline, the proportion contribution of each of the four risk-sectors to total caries risk for each participant was calculated. Applying these proportions to 24 (total number of text messages to be sent), the number of text messages on each risk-sector for each participant was determined. If, as a result of rounding, the sum of text messages to be sent was greater than 24, the number of ‘Circumstances’ messages was reduced because this risk-sector includes unlikely-to-be-changeable risk indicators. If, as a result of rounding, the total number was less than 24, the number of text messages in the risk-sector with the highest proportion was increased in order to highlight the highest risk-sector. If the participant had past root caries experience, the text message on root caries was always included. If the participant had a specific systemic disease, the text message on that disease was always included.

**Fig. 1:**
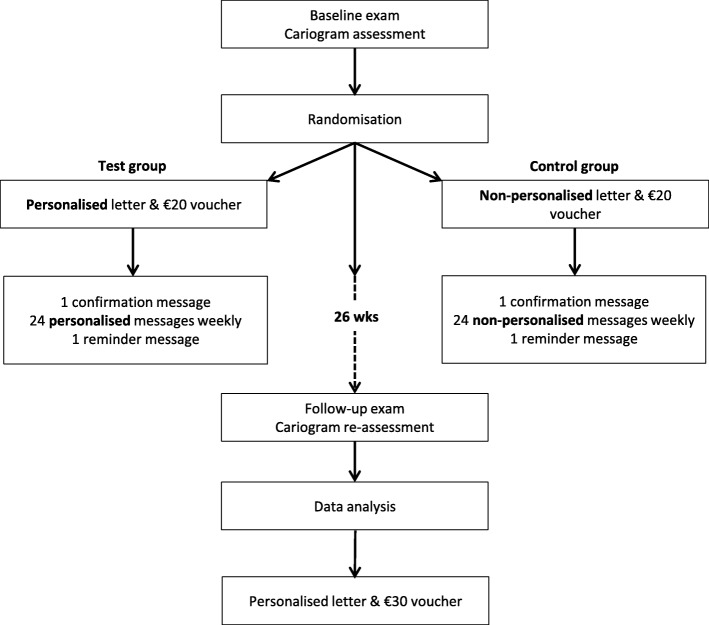
Workflow diagram. The similarity of interventions between the personalised and non-personalised groups was the sending of one letter and of 24 weekly text messages. As text messages were chosen by their priority ranking, the top ranking messages would have been sent to participants in both groups. If a participant was assigned into the personalised group, a staff (LF) posted a personalised letter which gave their ‘*chance of avoiding new cavities*’, their Cariogram chart results and advice relevant to their results with €20 vouchers as a gesture of thanks. The programmer was supposed to select text messages from each risk-sector in order of their priority ranking as detailed above for weekly sending to the participant. If a participant was assigned into the non-personalised group, LF posted general information on caries prevention cited from the Dental Health Foundation website [21] with additional information extracted from the Cariogram’s advices (non-personalised) in order that the letter volume was the same as for the personalised group and €20 vouchers a gesture of thanks. Then, the programmer was supposed to send each participant in the non-personalised group the predetermined, fixed set of 24 weekly text messages (the same six from each of the four risk-sectors with the highest priority ranking). The interventions for each group were administered between 26 April 2015 and 8 May 2016. With the €30 voucher at follow-up, we sent all participants and their dentists the results from both their baseline and follow-up CRAs plus their charts and personalised advice created by the Cariogram

For the bulk sending of text messages, the computer programmer used a web-based text messaging service (TextMagic, United Kingdom) [31] to send 24 educational text messages weekly [see Additional files 1 and 2]. Additionally, the programmer was supposed to send a welcome message asking each participant to send a reply as confirmation that we had their correct mobile number, and a final thank-you message reminding them to attend for their follow-up examination. We decided to send text messages between 5 and 6 pm on Sundays as this schedule was deemed most appropriate in the RoI context. Figure 1 presents a workflow diagram of the current study.

### Assessments and outcomes

To input the nine parameters of the Cariogram according to its manual [13], the dental practitioners examined and re-examined the participants using the case report form (CRF) and CRT® (Ivoclar Vivadent, Liechtenstein). The volume of stimulated saliva over 5 min was collected by chewing a paraffin pellet. The saliva was drooled into a disposable graduated test tube through a disposable funnel during the collection period. Participants completed their three-day food diary and study questionnaire at their homes and returned them via post to the OHSRC. The laboratory technician at the OHSRC incubated and read CRT® saliva tests. We extracted information from their CRF, CRT® saliva tests, three-day food diary and study questionnaire to score and assess the demographic factors (age, gender, smoking status, educational level, possession of smartphone and dental practice) and the Cariogram parameters (except ‘Clinical judgement’).

The pre-specified primary outcome measure was ‘*chance of avoiding new cavities*’ (0–100) from the Cariogram at follow-up. Note that a bigger ‘*chance of avoiding new cavities*’ indicates a lower total of the four risk-sector values (lower caries risk). The pre-specified secondary outcome measures were the seven biological risk parameters out of the ten risk parameters mentioned above: ‘Diet contents’ (salivary lactobacillus count with CRT® saliva test), ‘Diet frequency’ (frequency of fermentable carbohydrate intake), ‘Plaque amount’, ‘Mutans streptococci’, ‘Fluoride programme’, ‘Saliva secretion’ and ‘Saliva buffer capacity’.

This work was carried out at the OHSRC during a seven-month period (between 17 April 2015 and 8 November 2015) for the baseline data, and eight-month period (between 28 October 2015 and 19 July 2016) at follow-up. We obtained actual logs of sent text messages from TextMagic on 7 June 2017 [see Additional file 1]. From question number 13 (Q13) of the follow-up questionnaire, information on how many text messages were NOT understood (17–24, 9–16 or 1–8 messages) was extracted.

Before CRA commenced, the scoring of ‘Mutans streptococci’ and ‘Clinical judgement’ was adjusted, as we had found a lower score distribution of ‘Mutans streptococci’ and a lower risk distribution of ‘*chance of avoiding new cavities*’ among the first 52 participants compared to previous studies [17, 18] [see Additional file 4].

### Statistical analyses

From the baseline CRF and questionnaire, information on participant characteristics was extracted. For the primary analysis, we included all participants (*n* = 111) for the intent-to-treat (ITT) approach. For the per-protocol analysis, data deviations were calculated according to the actual message log and Q13 in the follow-up questionnaire. Duplicate (or more) messages which were accidentally sent to participants were excluded from the per-protocol analysis. Data deviations relating to time factor were ignored for the current paper. For secondary outcome measures (the seven risk parameters), Scores 0 and 1, and Scores 2 and 3 (if any) were combined as ‘lower score’ and ‘higher score’, respectively, in accordance with the Cariogram’s advice built into the software and the previous review paper [6]. The primary outcome was analysed using analysis of covariance (ANCOVA). The baseline value and age were included as covariates. Gender, Dental Practice and Group (personalised and non-personalised) were included as factors. The secondary outcomes were analysed using logistic regression models. The baseline values and age were included as covariates. Gender and Group (personalised and non-personalised) were included as factors. Dental Practice could not be included as the number of categories resulted in quasi-separation in logistic regression models. We set the significance level of 5% (two-sided). We utilised SAS, Version 9.4 (SAS Institute Inc., Cary, NC).

## Results

**Fig. 2:**
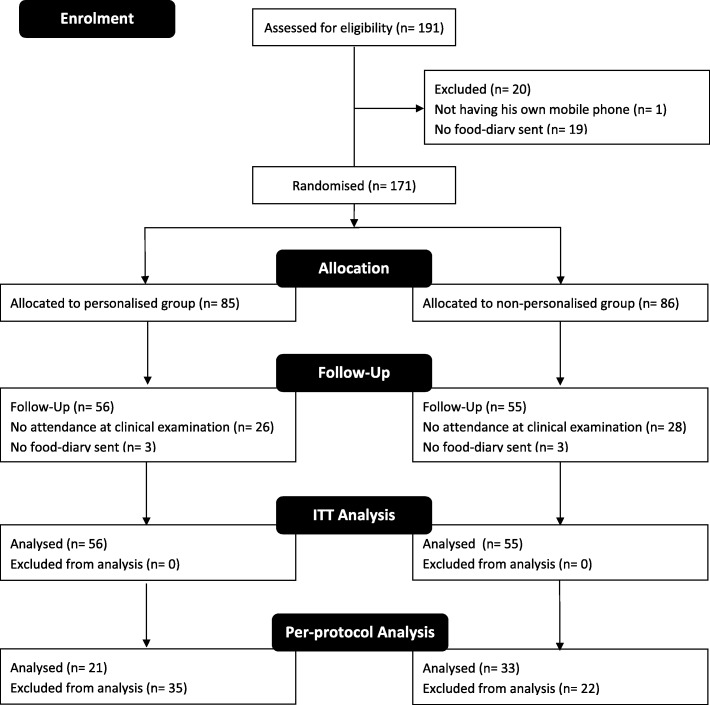
Subject disposition/CONSORT flow diagram. Before randomisation, one participant who did not have his own mobile-phone and 19 participants who did not return their baseline three-day food diary to the Oral Health Services Research Centre (OHSRC) were excluded. Of the 171 participants included in the study, 26 out of 85 in the personalised group and 28 out of 86 in the non-personalised groups did not attend their follow-up examination, and three participants in each group did not return their three-day food diary to the OHSRC. As a result, 56 and 55 participants in the personalised and non-personalised groups, respectively, were analysed. Due to unexpected protocol violations, only two and nine participants in the personalised and non-personalised groups, respectively received their planned educational messages within the scheduled 24-week time period [see Additional file 1]. For this reason, for the per-protocol analysis, we ignored time factor and priority ranking violations and allowed two-message deviations. In total, 21 and 33 participants were included for per-protocol analysis in the personalised and non-personalised groups, respectively

Figure 2 summarises the participant flow through the study. The dental practitioners recruited the participants between 25 February 2015, and 28 September 2015 at baseline, and re-examined them between 14 October 2015 and 19 May 2016 at follow-up. The cut-off for receiving the three-day food diary and questionnaire was 02 November 2015 at baseline and 16 June 2016 at follow-up. The follow-up CRA was finished on 19 July 2016 as dentists could get no more patients to attend for follow-up.

The demographic characteristics of the sample are presented in Table 1. While there were more females than males in both groups, the proportion of females in the personalised group was lower than in the non-personalised group. Two dental practitioners (Dentists A and F) lost all their patients at follow-up. The distribution of participants for the six remaining dentists was highly uneven.

**Table 1:** Demographic characteristics of the sample

Variables	Participants at randomisation		Participants at follow-up	
	Personalised	Non-personalised	Personalised	Non-personalised
	(*n* = 85)	(*n* = 86)	(*n* = 56)	(*n* = 55)
Age, y, n (%)				
< 20	7 (8.2)	5 (5.8)	1 (1.8)	2 (3.6)
20–29	12 (14.1)	22 (25.6)	7 (12.5)	7 (12.7)
30–39	28 (32.9)	25 (29.1)	18 (32.1)	17 (30.9)
40–49	21 (24.7)	18 (20.9)	20 (35.7)	16 (29.1)
50–59	12 (14.1)	10 (11.6)	6 (10.7)	8 (14.5)
60–69	5 (5.9)	6 (7.0)	4 (7.1)	5 (9.1)
Mean (SD)	38.9 (12.8)	37.3 (13.0)	40.9 (11.8)	41.2 (12.3)
Median (min. to max.)	37 (17–69)^a^	36 (18–69)^a^	40 (19–69)	40 (19–69)
Gender, *n* (%)				
Female	54 (63.5)	64 (74.4)	34 (60.7)	41 (74.5)
Educational level, *n* (%)				
Less than third level	50 (58.8)	49 (57.0)	31 (55.4)	29 (52.7)
Third level and more	31 (36.5)	28 (32.6)	25 (44.6)	20 (36.4)
Still in education	1 (1.2)	6 (7.0)	0 (0.0)	3 (5.5)
Missing	3 (3.5)	3 (3.5)	0 (0.0)	3 (5.5)
Smoking status, *n* (%)				
Non-smoker	57 (67.1)	62 (72.1)	43 (76.8)	42 (76.4)
Smoker	28 (32.9)	24 (27.9)	13 (23.2)	13 (23.6)
Smart phone, *n* (%)				
Non-possession	15 (17.6)	15 (17.4)	12 (21.4)	12 (21.8)
Possession	64 (75.3)	64 (74.4)	41 (73.2)	40 (72.7)
Missing	6 (7.1)	7 (8.1)	3 (5.4)	3 (5.5)
DMFS				
Mean (SD)	31.0 (19.4)	31.7 (18.6)	32.6 (20.2)	34.9 (19.0)
Median (min. to max.)	33 (0–106)	29.5 (0–66)	33 (1–106)	33 (0–66)
Dental practice, *n* (%)				
A	1 (1.2)	1 (1.2)	0 (0.0)	0 (0.0)
B	8 (9.4)	14 (16.3)	7 (12.5)	11 (20.0)
C	9 (10.6)	9 (10.5)	9 (16.1)	9 (16.4)
D	44 (51.8)	43 (50.0)	32 (57.1)	32 (58.2)
E	8 (9.4)	7 (8.1)	4 (7.1)	2 (3.6)
F	1 (1.2)	1 (1.2)	0 (0.0)	0 (0.0)
G	6 (7.1)	3 (3.5)	1 (1.8)	1 (1.8)
H	8 (9.4)	8 (9.3)	3 (5.4)	0 (0.0)

*SD* Standard deviation, *DMFS* Decayed missing filled tooth surfaces

^a^Since one dentist did not comply with the inclusion criteria for age, one 17-year-old patient and one 18-year-old patient were included in the personalised group and one 18-year-old patient in the non-personalised group. All of them did not complete the study. See the ‘Subjects’ section for the pre-determined inclusion criteria

Table 2 shows a comparison of the number of text messages from the four risk-sectors, both assigned and actually sent, between the personalised and non-personalised groups. In Q13, two participants answered they did not understand 17–24 messages and another two participants answered they did not understand 1–8 messages. One participant wrote in the questionnaire that she did not receive any text messages.

**Table 2:** Assigned and actually sent text messages by each risk-sector to the personalised and non-personalised groups

Number of text messages	Personalised Group				Non-personalised Group			
	Diet	Bacteria	Susceptibility	Circumstances	Diet	Bacteria	Susceptibility	Circumstances
Assigned messages								
Sum	401	504	264	175	330	330	330	330
Mean (SD)	7.2 (2.9)	9.0 (3.4)	4.7 (4.2)	3.1 (1.7)	6.0 (0.0)	6.0 (0.0)	6.0 (0.0)	6.0 (0.0)
Median	7	9	3	3	6	6	6	6
Range	1–13	3–16	2–18	0–7	6–6	6–6	6–6	6–6
Actually sent messages^a^								
Sum	340	422	217	146	287	313	292	294
Mean (SD)	6.1 (3.0)	7.5 (3.4)	3.9 (3.2)	2.6 (1.6)	5.2 (0.9)	5.7 (0.6)	5.3 (1.1)	5.3 (0.7)
Median	6	7	3	2.5	5	6	6	5
Range	0–12	0–14	0–16	0–6	3–6	3–7	2–6	3–6

*SD* Standard deviation

^a^Duplicates (or more) were counted as one

For the primary analysis, with the ITT approach, means (standard deviation) of ‘*chance of avoiding new cavities*’ were 46.2 (± 19.6) in the personalised group (*n* = 56) and 42.8 (± 22.0) in the non-personalised group (*n* = 55) (Table 3). The ANCOVA showed no statistically significant difference between the two groups (mean difference (95% confidence interval (CI)) = 0.7 (− 5.5, 6.9), *p* = 0.820). For the secondary outcome, with the ITT approach, only the stimulated saliva flow factor showed a personalised intervention effect, *p* = 0.036, odds ratio (OR) = 0.3 (95% CI = 0.1, 0.9).

**Table 3:** Intent-to-treat (ITT) analysis: primary and secondary outcomes between the personalised and non-personalised groups

ITT analysis	Group			
	Personalised (*n* = 56)	Non-personalised (*n* = 55)		*p* value
Primary outcome (‘chance of avoiding new cavities’)			Mean difference (95% CI)	
Baseline				
mean (SD)	39.3 (20.2)	36.5 (23.4)		
median (min. to max.)	37.5 (6 to 81)	31.0 (3 to 94)		
Follow-up			0.7 (−5.5, 6.9)	*p* = 0.820
mean (SD)	46.2 (19.6)	42.8 (22.0)		
median (min. to max.)	44.5 (8 to 83)	41.0 (9 to 93)		
Secondary outcome (number (%) of participants with Score 0, 1)			Odds ratio (95% CI)	
‘Diet frequency’				
Baseline	39 (69.6)	36 (65.5)		
Follow-up	47 (83.9)	43 (78.2)	0.8 (0.3, 2.3)	*p* = 0.663
‘Diet contents’				
Baseline	27 (48.2)	30 (54.5)		
Follow-up	27 (48.2)	30 (54.5)	1.0 (0.4, 2.6)	*p* = 0.945
‘Plaque amount’				
Baseline	25 (44.6)	19 (34.5)		
Follow-up	31 (55.4)	33 (60.0)	1.7 (0.7, 3.9)	*p* = 0.247
‘Mutans streptococci’				
Baseline	34 (60.7)	24 (43.6)		
Follow-up	36 (64.3)	31 (56.4)	1.1 (0.4, 2.6)	*p* = 0.917
‘Fluoride programme’^a^				
Baseline	55 (98.2)	51 (92.7)		
Follow-up	56 (100.0)	54 (98.2)		*p* = 0.941
‘Saliva secretion’				
Baseline	45 (80.4)	40 (72.7)		
Follow-up	51 (91.1)	41 (74.5)	0.3 (0.1, 0.9)	*p* = 0.036*
‘Saliva buffer capacity’				
Baseline	54 (96.4)	51 (92.7)		
Follow-up	45 (80.4)	40 (72.7)	0.8 (0.3, 2.1)	*p* = 0.653

*p < 0.05

ITT intent-to-treat, SD Standard deviation, CI Confidential interval

^a^Model fit was questionable – odds ratio estimates unreliable

The primary outcome is a comparison of ‘*chance of avoiding new cavities*’ calculated by the Cariogram. The secondary outcome measures are the seven biological risk parameters out of the ten risk parameters in the Cariogram. Scores 0 and 1, and Scores 2 and 3 (if any) are combined as ‘lower score’ and ‘higher score’, respectively. The table indicates number (%) of participants with ‘lower score’

For the per-protocol analysis, there was no statistically significant difference between the two groups (mean difference (95% CI) = 4.0 (− 5.6, 13.5), *p* = 0.410) (Table 4). For the secondary outcomes, logistic regression estimates were not reliable due to the small sample size for the per-protocol analysis. There was no harm or unintended effects in either group.

**Table 4:** Per-protocol analysis: primary and secondary outcomes between the personalised and non-personalised groups

Per-protocol analysis	Group			
	Personalised (*n* = 21)	Non-personalised (*n* = 33)	Mean difference (95% CI)	*p* value
Primary outcome (‘chance of avoiding new cavities’)				
Baseline				
mean (SD)	36.7 (18.6)	29.4 (20.6)		
median (min. to max.)	37 (11 to 67)	26 (3 to 83)		
Follow-up			4.0 (−5.6, 13.5)	*p* = 0.410
mean (SD)	44.6 (18.4)	35.0 (20.6)		
median (min. to max.)	39 (16 to 83)	32 (9 to 84)		
Secondary outcome (number (%) of participants with Score 0, 1)^a^				
‘Diet frequency’				
Baseline	12 (57.1)	21 (63.6)		
Follow-up	18 (85.7)	25 (75.8)		
‘Diet contents’				
Baseline	10 (47.6)	14 (42.4)		
Follow-up	8 (38.1)	13 (39.4)		
‘Plaque amount’				
Baseline	8 (38.1)	9 (27.3)		
Follow-up	12 (57.1)	18 (54.5)		
‘Mutans streptococci’				
Baseline	9 (42.9)	8 (24.2)		
Follow-up	12 (57.1)	12 (36.4)		
‘Fluoride programme’				
Baseline	21 (100.0)	30 (90.9)		
Follow-up	21 (100.0)	32 (97.0)		
‘Saliva secretion’				
Baseline	20 (95.2)	23 (69.7)		
Follow-up	21 (100.0)	24 (72.7)		
‘Saliva buffer capacity’				
Baseline	21 (100.0)	31 (93.9)		
Follow-up	18 (85.7)	27 (81.8)		

SD Standard deviation, CI Confidential interval

^a^Logistic regression estimates were not reliable due to the small sample size

The primary outcome is a comparison of ‘*chance of avoiding new cavities*’ calculated by the Cariogram. The secondary outcome measures are the seven biological risk parameters out of the ten risk parameters in the Cariogram. Scores 0 and 1, and Scores 2 and 3 (if any) are combined as ‘lower score’ and ‘higher score’, respectively. The table indicates number (%) of participants with ‘lower score’

## Discussion

This study tried to compare the effects of personalised versus non-personalised interventions via text messaging on caries risk in an economically disadvantaged adult population. The reason for selecting text messaging as an intervention method was that it has enormous potential for conducting personalised approaches to disease prevention and management [7]. CRA was selected as a primary outcome, because caries risk reduction could be observed in the six-month study period [32]. Among various tools for caries risk assessment, the Cariogram was selected because it is validated [6] and shows the risk profile graphically for patient education [13]. The null hypothesis in regard to the primary outcome for both ITT and per-protocol analyses was not rejected. However, as the MCID was included in the 95% CI for the per-protocol analysis, replication studies will be worth conducting.

The reason for considering one- or two-message deviations as acceptable for the per-protocol analysis was that an error of less than three messages occurred in the rounding procedure for deciding the number of text messages within each risk-sector. The reason the sample size of the personalised group (*n* = 21) was considerably smaller than that of the non-personalised group (*n* = 33) is likely because the programmer continued to use the failed computer program. It was used to display personalised combinations of text messages even after he found that the program could not properly be programmed to send text messages to TextMagic in the third week [see Additional file 1]. For the non-personalised group, the computer program was not used to select text messages as the non-personalised group received a predetermined, fixed set of text messages. Thus, the personalised group was more affected, being subject to multiple errors.

The stimulated saliva flow parameter was significantly influenced in the personalised group for the ITT analysis, although the number of sent text messages on the ‘Susceptibility’ sector was not many. For the per-protocol analysis, all of the 21 participants had the lower risk score for this parameter. In another paper using the baseline data (*n* = 159), we found that knowledge of saliva factors as being a caries risk was quite low in this Irish population [15]; approximately 70% of the respondents did not know that a reduced amount of saliva is a caries risk factor. From these results, providing information on risk factors/indicators they are not already familiar with would have greater impact when informing the patient of the results of his/her individual CRA. Yet, the positive change in stimulated saliva flow at the follow-up examination may not indicate a true increase of saliva flow in daily life, as participants in the personalised group may have tried spitting more saliva, possibly because they learned from their personalised letter that they did not have enough saliva, and from their text messages that it is an important factor.

One reason for the unclear difference of ‘*chance of avoiding new cavities*’ between the two groups may be the sensitive design of the current study. The non-personalised group were sent the six highest prioritised text messages for each risk-sector, which would include the text messages that would also be chosen for the personalised group depending on their risk profile. Also, the non-personalised letter contained information from the Cariogram’s advices (non-personalised) in order to have the same letter volume as the personalised group. As a result, the interventions to the personalised participants were not markedly different from those for the non-personalised participants, unless a participant had a prominent risk profile. On the other hand, in a randomised controlled trial for smoking cessation sending mobile-phone text messages to both test and control groups, all text messages for the test group were personalised ones related to quitting and all text messages to the control group were clearly unrelated to quitting [33]. In another study for weight loss, although there was some overlapping information between the test and control groups, the test group received personalised mobile-phone text messages two to five times daily plus other services whereas the control group received the print material only once a month [34]. Our study did not have such clear contrast in interventions between the test and control groups. Instead, we designed the current study with a much narrower interest that aimed to look into an effect of a personalised combination of text messages based on individual CRA, while keeping other conditions as equal as possible between the test and control groups. While it is likely that the mHealth intervention benefited both groups for caries risk reduction, our study design cannot fully validate this as the patients, examiners (dentists) and assessors (the laboratory technician and MN) might unconsciously wish and evaluate better at follow-up than at baseline.

When this study was designed, only 57% of mobile-phone customers owned a smartphone in the RoI [35]; we estimated this percentage would be even lower in a disadvantaged group and opted for short text messaging instead of smartphone messaging. Since an exponential rise in smartphone use was expected in the RoI, we included the question on smartphone ownership in the CRF for a future study. The response to this question indicated that approximately three-quarters of the participants already had a smartphone. Therefore, services via smartphone would be the choice for mHealth today, even in a disadvantaged population in RoI. Some smartphone instant messaging applications signal the sender when the receiver has read a message; information on whether the participant opens the message or not is useful. Artificially intelligent chatbots will easily enable an interactive approach with participants, and may give greater motivation to participants.

The current study has limitations on its generalisability. The response rate was low and may cause selection bias. The participants who dropped out tended to be younger, with less than third level education, smokers, smartphone owners and with less mean number of Decayed Missing Filled Tooth Surfaces (DMFS) than ones at follow-up. Although the sample size was slightly underpowered, the *p* values were not near the significant level. Therefore, there may not be a major risk of Type 1 or 2 errors with the similarity of data in both groups. Even though we gave a rather high compensation (€50) to encourage the disadvantaged population, results showed that 79 out of 191 participants (49%) did not comply with the study procedure. The reasons may be (1) that this population is difficult to keep compliant, (2) that reminder text messages for the follow-up examination were actually not sent to 15 participants (60% of them did show for the follow-up examination) [see Additional file 1] and (3) that Dentist H changed work place during the period of follow-up examinations. Another limitation is that the time frame varied largely from individual to individual. One-fourth of the participants posted the three-day food diaries to the OHSRC more than 2 months after the intervention finished. The effect of educational text messages may be decreased when there are lengthy time delays to have an effect, as the long-term effect of mHealth is still uncertain [36, 37].

The current study did not investigate the effect of text messages on actual disease level (caries incidence, gingivitis, periodontal disease and so on) but only on CRA. When the long-term effect of mHealth for oral health can be investigated in the future, the actual disease level should be of interest. In the current study, the dental practices did not routinely perform patient education based on CRA. In reality, it would be preferable that personalised patient education is performed by the dental practice and that personalised mHealth is used as an auxiliary measure to compensate and re-enforce the patient education at practice and to engage the patients.

## Conclusions

The null hypothesis that no difference would exist between personalised and non-personalised interventions among economically disadvantaged adults was not rejected. However, it is worth exploring further the potential of mobile-devices for individual caries risk reduction.

## Additional files

Additional file 1: The protocol violations in more detail. (PDF 86 kb)

Additional file 2: Details on the allocation concealment. (PDF 104 kb)

Additional file 3: Twelve examples of text messages on the four risk-sectors. (PDF 49 kb)

Additional file 4: Scoring each risk parameter of the Cariogram in the current study. (PDF 168 kb)

## Abbreviations

ANCOVA: Analysis of Covariance
CI: Confidence Interval
CONSORT: Consolidated Standards of Reporting Trials
CRA: Caries Risk Assessment
CRF: Case Report Form
DMFS: Decayed Missing Filled Tooth Surfaces
ITT: Intent-to-treat
MCID: Minimal Clinically Important Difference
mHealth: Mobile Health
OHSRC: Oral Health Services Research Centre
OR: Odds Ratio
PSAP: Promoting State-of-the-Art risk assessment for the Prevention of dental caries and periodontal disease
Q: question number
RoI: Republic of Ireland
SD: Standard Deviation
UCC: University College Cork
