# Supplementary material for: The effect of mobile personalised texting versus non-personalised texting on the caries risk of underprivileged adults: a randomised control trial
Source: BMC Oral Health. 2019 Mar 12;19:44. doi: 10.1186/s12903-019-0729-1 (PMC6417196; doi:10.1186/s12903-019-0729-1)
Supplement: Supplementary file 1 — The protocol violations in more detail. (PDF 86 kb) [file 12903_2019_729_MOESM1_ESM.pdf]

## Additional file 1

## The protocol violations in more detail

Approximately one year after the end of our study intervention, during the writing of this manuscript, we found critical protocol deviations caused by the computer programmer employed for the study and discovered that he had concealed facts in the attempt to mislead the research team of his failings. Fortunately, the web-based text messaging service which the programmer used (TextMagic, United Kingdom) [1] keeps actual logs of incoming and outgoing text messages and we were able to download these logs on 7 June 2017. Examination of the TextMagic log revealed the extent of deviations from project protocol. Differences in the number of text messages actually sent and the number assigned for sending by the four risk-sectors are presented in Table 2 of the main text.

Some bugs and glitches are inevitable in mobile health (mHealth) research and it is recommended to perform internal and external testing prior to the beginning of an mHealth intervention [2]. The TextMagic log shows that any text message based on caries risk assessment was tried by the programmer before intervention commenced in our research project. During the intervention, there were two chances for us to prevent further data deviations. Two participants separately gave us information on messaging inconsistencies at the beginning and in the middle of the intervention period. Although the programmer responded that the issue would be resolved for the first participant and that messages to the second participant were sent with no issue, the actual log shows that messaging failures were occurring every week from the beginning to the end of the 55-week intervention period, without being fixed. It is regrettable that we did not exploit these two opportunities to rectify any deviations, trusting instead in the programmer's expertise and integrity.

One important lesson for the fidelity of the intervention is the importance of having a third person watch the intervention process. In this case, we could have allocated a third person to

sign in to TextMagic and examine actual logs every week. It is also recommended to add multiple dummy recipients who monitor text messages received during the intervention period. Another lesson is to always evaluate the pros and cons when you change the situation. We were supposed to use the services of Rapport Builder® (Oral Care Inc., Japan) [3], which inspired the current study. The merits of changing from Rapport Builder® to the student programmer in the School of Computer Science and Information Technology within our university were (1) that communication could be done locally and (2) that we could save the allocated budget to Rapport Builder®. We had had discussions about contingencies in this research project, however we never discussed the demerits of this change and precautions for the involvement of a student programmer in the research team. Precautions should have included the drawing up of an official contract with technical specifications for his participation, a clear terms of reference outlining the responsibility of the student as a research team member, and the provision of appropriate training on Good Research Practice [4].

In mHealth research, it is acceptable to ask the assistance of an IT undergraduate student as part of his/her bachelor project under supervision. However, it should be noted that this is a rather high-risk bet. The student in his/her final year may even manipulate data with his/her IT expertise without fulfilling his/her reporting obligation to his/her supervisor or realising the gravity of what they are doing. And once they have graduated, there is no sanction for their actions. Therefore, if there is a critical failure in his/her bachelor project at the last stage, it is no surprise that a student will attempt whatever is necessary for them to achieve graduation. On the other hand, a programmer employed by a company would be held responsible for their actions and be subject to punishment and condemnation, which makes the risk of data manipulation lower. In addition, he/she and the company would suffer from a black mark on their professional reputation. Although hiring the student programmer was cheaper than contracting Rapport Builder®, the irreversible loss this change cost the current study cannot

be counted.

Use of mHealth for caries prevention was a frontier topic four years ago when this project started. Thus, we had little knowledge of how to work with an IT expert, who would be at the core of the research project. Without enough IT expertise, we were naïve and treated the programmer as a sacred cow. Indeed, no one in our research team except the programmer knew that TextMagic provides various functions, which easily allows for the checking of message failures, until we had to contact TextMagic to verify discrepancies found in the programmer's log and saw these functions. Consequently, from this bitter experience we learned first-hand the vulnerability of mHealth to programmer manipulation. At the same time, rigorous detail logs are automated and easily available thanks to digital technology, which is a great advantage for mHealth research. From the TextMagic log, we could grasp exactly what was happening with the assigned 4,446 (= 26*171) text messages and analyse the factual data.

It is important to use this experience to raise the overall level of scientific integrity [5], especially for this rapidly emerging mHealth field which has huge potential [6]. To prevent the repeating message failures, which was never fixed by the student programmer, the feasibility of the computer programme designed for this study is going to be investigated by an expert as far as our budget allows. We must fulfil accountability to all people involved in this research project about protocol deviations and the resultant findings for the integrity of the study. Especially, to the two participants mentioned above, we appreciate their feedback that became important clues in our investigation to reach closer to the truth.

## References

1. TextMagic Ltd. https://www.textmagic.com/. Accessed 10 November 2018.
2. Ben-Zeev D, Schueller SM, Begale M, Duffecy J, Kane JM, Mohr DC. Strategies for mHealth research: lessons from 3 mobile intervention studies. Adm Policy Ment Health.

2015;42:157-67.

3. Oral Care Inc. About Rapport Builder. https://www.ocm-navi.jp/about/about.html. Accessed 10 November 2018 (In Japanese).
4. Smith AJ. Research integrity and scientific misconduct. J Dent Res. 2008;87:197.
5. Smith R. Research misconduct: the poisoning of the well. J R Soc Med. 2006;99:232-7.
6. Kay M, Santos J, Takane M. mHealth: new horizons for health through mobile technologies. World Health Organization. 2011;64:66-71.
