# Supplementary material for: The effect of mobile personalised texting versus non-personalised texting on the caries risk of underprivileged adults: a randomised control trial
Source: BMC Oral Health. 2019 Mar 12;19:44. doi: 10.1186/s12903-019-0729-1 (PMC6417196; doi:10.1186/s12903-019-0729-1)

**Additional file 2**

**Details on the allocation concealment**

Allocation was concealed using sealed, opaque, sequentially numbered envelopes. The envelopes were handed out by a staff (LF) who was not involved in assessing data. LF enrolled and assigned the participants under the direction of the statistician. MN prepared personalised and non-personalised letters for each participant and passed them to LF together with their '*chance of avoiding new cavities*' scores for their stratification into the five groups. LF informed the person who was to send the text messages (computer programmer) of each participant's mobile number and which group they were in. Apart from LF, the computer programmer and the participants, all involved in the research project were blinded. Participants were randomised between 17 April 2015, and 12 November 2015. After all data entry was completed, the group assignments were revealed on 16 August 2016.

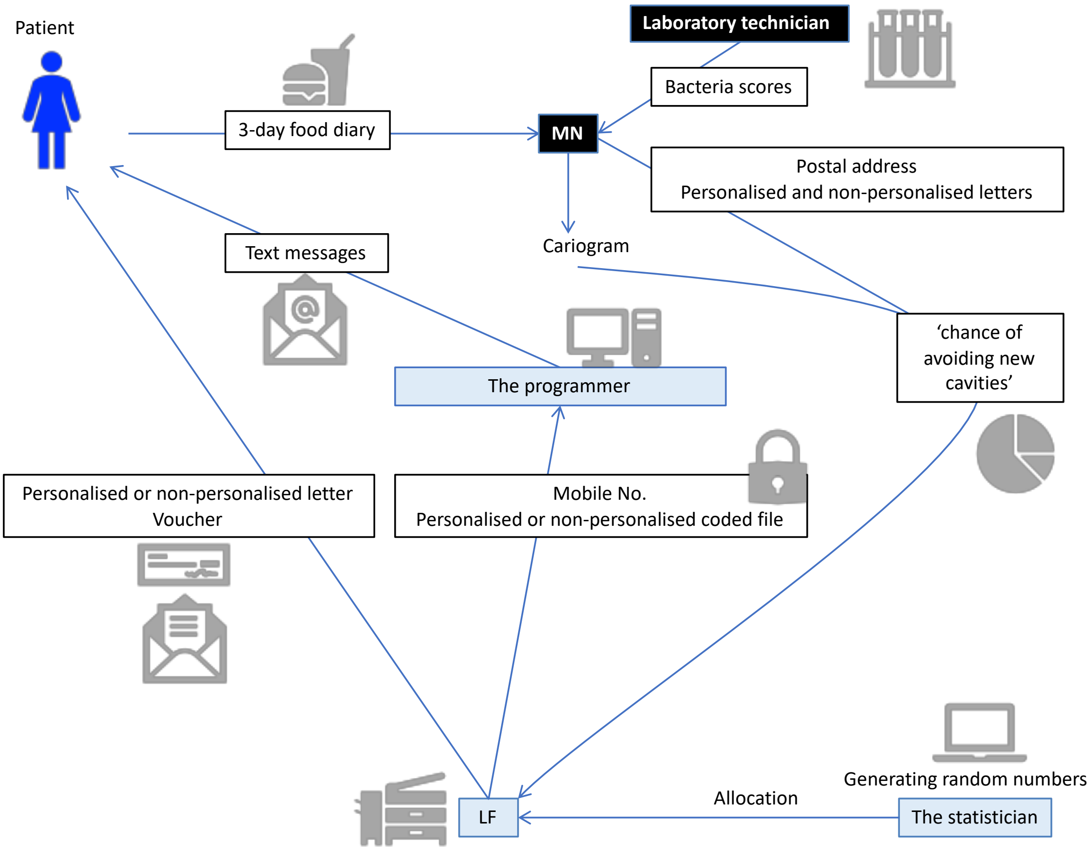

Supplement: Supplementary file 2 — Details on the allocation concealment. (PDF 104 kb) [file 12903_2019_729_MOESM2_ESM.pdf]
