# Supplementary material for: The effect of mobile personalised texting versus non-personalised texting on the caries risk of underprivileged adults: a randomised control trial
Source: BMC Oral Health. 2019 Mar 12;19:44. doi: 10.1186/s12903-019-0729-1 (PMC6417196; doi:10.1186/s12903-019-0729-1)
Supplement: Supplementary file 3 — Twelve examples of text messages on the four risk-sectors. (PDF 49 kb) [file 12903_2019_729_MOESM3_ESM.pdf]

Additional file 3

Twelve examples of text messages on the four risk-sectors

Risk-sector	Text messages
**Diet**	"Hi! Within 2 hrs of bedtime is the worst time for sugar-sweetened snacks/drinks. As we don't make much saliva during sleep, the acid attack can last many hours."
	"Hi! Prevent tooth decay by making smart & healthy food choices: foods & drinks high in sugar can lead 2 tooth decay & weight gain. Eat smart, stay healthy!"
	Hi! Give your teeth a break! Leave at least 2 hours between every meal or snack! That way your teeth have time to heal from acidic effects of food.
**Bacteria**	Hi! Brush thoroughly w/ fluoride toothpaste 2 minutes twice a day, more often if ur dentist recommends! Small circular movements r good 2 clean ur teeth.
	The thin sticky film or 'furry' feeling that forms on ur teeth contains bugs, It's called a biofilm. Gunk that clogs kitchen & bathroom drains is biofilm too!
	Lots of bugs live in the mouth & some produce acids. Acids attack ur teeth, causing cavities. Don't leave it there! Cleaning teeth morning & night really helps.
**Susceptibility**	Hi Tooth study here! Fluoride in your toothpaste helps to strengthen and protect teeth, which can reduce tooth decay in adults and children.
	Hi Tooth study here! Adults should use a toothpaste that contains at least 1450ppm of fluoride twice a day to prevent decay.
	Hi Tooth study here! During sleep, saliva flow is very low & acid attacks to the tooth surface can last for many hours. Best to sleep & not eat in bed!
**Circumstances**	Hi! Over the years, fillings may weaken & tend to fracture & leak around the edges. Visit your dentist regularly for professional cleanings & oral examination!
	Hi Tooth study here! Regular dental visits help prevent decay. Going to the dentist for prevention regularly can make life more pleasant.
	Prevention is better than cure! Visit your dentist regularly: your dentist will spot problems earlier, helping u care for ur teeth.
