# Supplementary material for: The effect of mobile personalised texting versus non-personalised texting on the caries risk of underprivileged adults: a randomised control trial
Source: BMC Oral Health. 2019 Mar 12;19:44. doi: 10.1186/s12903-019-0729-1 (PMC6417196; doi:10.1186/s12903-019-0729-1)
Supplement: Supplementary file 4 — Scoring each risk parameter of the Cariogram in the current study. (PDF 168 kb) [file 12903_2019_729_MOESM4_ESM.pdf]

Additional file 4

Scoring each risk parameter of the Cariogram in the current study

'Caries experience'

First, the number of decyed missing filled tooth surfaces (DMFS) was recorded both at dentinal and at the cavitated dentine level. Cavitated DMFS (D_{3c}MFS) and non-cavitated DMFS (D_{3vc}MFS)[1] values were calculated from the case report form (CRF). Three surfaces were counted as missing for teeth that were missing due to any reason in accordance with the Oral Health of Irish Adults 2000–2002 [1].

Additional file Table 1 Reference values

		16-24 years	35-44 years	65+ years
**D_{3c}MFS index**	25% cut off scores	3	23	58
	75% cut off scores	18	48	96
**D_{3vc}MFS index**	25% cut off scores	4	24	59
	75% cut off scores	19	49	96

The 'Caries experience' parameter is a relative score with reference to local epidemiological data [2]. The current study used the latest available Irish adult data [1] as its reference. Additional file Additional file Table presents the cut-off scores of the D_{3c}MFS and D_{3vc}MFS values from the reference data for the 25th and 75th percentiles by age group (16–24, 35–44, 65+). Cut-off scores for the 25th and 75th percentiles at ages 20-, 40- and 70-years were plotted.

[1] D_{3vc}MFS includes non-cavitated where there was a definite shadow under the enamel, indicating the presence of dental caries that had progressed to dentine, but cavitation had not yet occurred.

With the assumption that the $D_{3c}MFS$ and $D_{3vc}MFS$ values increase in a straight line according to age, straight lines between the scores at 20 and 40 years of age and between 40 and 70 years of age for the 25th and 75th percentiles were drawn. If a medical-card patient's $D_{3c}MFS$ index fell below the 25th percentile line, the a medical-card patient was scored as Score 1 (better than normal). If a medical-card patient's $D_{3c}MFS$ index fell above the 75th percentile line, the medical-card patient was scored as Score 3 (worse than normal). If a medical-card patient's $D_{3c}MFS$ index lay between the 25th and 75th percentile lines, the medical-card patient was scored as Score 2 (normal for age group). If the $D_{3c}MFS$ index of the medical-card patient fell on one of the lines, the worse score was taken. The $D_{3vc}MFS$ index was referenced in the same manner. If the medical-card patient had > 2 active root caries lesions or > 2 enamel lesions, the medical-card patient was given Score 3. Score 0 means that the patient was caries free and had no restorations.

'Related diseases'

General diseases or conditions which can directly or indirectly influence the caries process, were listed as follows [2]:

- any autoimmune disease (e.g. Sjögren's syndrome)
- diabetes mellitus
- anorexia nervosa
- visually impaired
- any manual dexterity which might cause them difficulties with cleaning their teeth properly
- any disease which requires continuous medication that affect their saliva secretion
- any condition requiring radiation to the head-neck region.

Score 0 was given for patients with none of the general diseases above (no disease). Score 1 was given if there any of the general diseases above was present (mild degree). The Cariogram Manual stated Score 2 should be given if the patient was bedridden or may need continuous medication (severe degree, long-lasting). Because medical-card patients taking part in the current study were not bedridden and the definition of 'long-lasting' was unclear, Score 2 was considered as not applicable.

'Diet contents'

Salivary lactobacillus count with CRT® saliva test (Ivoclar Vivadent, Liechtenstein) was used as an indicator of the 'diet contents' parameter [2]. Although retention areas, open cavities or bad fillings could contribute to a high LB score [2], these conditions were not considered in the current study. This parameter was scored using the manufacture's chart. Scores 0 and 1 were < 10^5 colony forming units (CFU)/ml saliva. Scores 2 and 3 were ≥ 10^5 CFU/ml saliva. The distinction between Scores 0 and 1 and between Scores 2 and 3 were made according to the manufacture's chart. The interpretation of scores was as follows:

Score 0: very low fermentable carbohydrate

Score 1: low fermentable carbohydrate, 'non-cariogenic' diet

Score 2: moderate fermentable carbohydrate

Score 3: high intake of fermentable carbohydrate.

'Diet frequency'

On their three-day food diary, the medical-card participant wrote down when and what he/she had eaten and what time their bedtime was for three days[2]. The mean intake of fermentable

[2] Although the medical-card patients were asked to record food diary "*during three ordinary days including a weekend day*", some medical-card patients in the current study did not comply with including two ordinary days and one weekend day.

carbohydrates per day was calculated. Dietary sugars (sucrose, glucose and fructose), cooked starches and sucralose were included in the basic count of fermentable carbohydrates. Although strictly speaking vegetables have natural sugars, they were not counted as part of fermentable carbohydrate intake because some of the educational text messages encouraged eating vegetables rather than sugary foods as snacks.

When the medical-card patient did not write their bedtime and the medical-card patient had fermentable carbohydrates at 10 pm or later, one intake count was added. When the medical-card patient wrote their bedtime and had fermentable carbohydrates within one hour before bedtime, one intake count was also added. The scores for this parameter are as follows:

Score 0: 0–3.0 times/day (very low diet intake frequency)

Score 1: 3.3–5.0 times/day (low diet intake frequency)

Score 2: 5.3–7.0 times/day (high diet intake frequency)

Score 3: ≥ 7.3 times/day (very high diet intake frequency).

‘Plaque amount’

Dental practitioners recorded a single score from 0 to 3, as defined in the Cariogram Manual [2], based on their clinical impression of each patient. The scores for ‘plaque amount’ are as follows:

Score 0: extremely good oral hygiene

Score 1: good oral hygiene

Score 2: less than good oral hygiene

Score 3: poor oral hygiene.

The decision to apply these adjustments was made on 16 April 2015, just before risk assessment and randomisation were performed for the first patient.

‘Mutans streptococci’

Salivary mutans streptococci (MS) count with CRT® was scored using the manufacturer’s chart which says Scores 0 and 1 were < 10^5 CFU/ml saliva and Scores 2 and 3 were ≥ 10^5 CFU/ml saliva. Note that the Cariogram was originally designed to use Dentocult® saliva test kits [2]. According to the CRT® instruction, CRT® bacteria correlates with the Dentocult® system; however, CRT® MS reacts more sensitively and is able to detect even low bacterial count. Both tests have a model chart with four pictures assessing the density of CFU/ml saliva. The distribution of MS in the current study showed much lower risk than shown by other studies [3-5] and clinical data from two Japanese dental practices using Dentocult SM® (Oral Care Inc., Tokyo) (Additional file Table 2), although the current study population was expected to be economically disadvantaged (i.e. a high-risk group). Therefore, Score 0 was rounded up to Score 1 and Score 2 was rounded up to Score 3 in the current study.

Additional file Table 2 Distribution of CRT Bacteria® (MS) Score compared to other data using Dentocult SM® (%)

Data source	*n*†	Score 0	Score 1	Score 2	Score 3
**CRT Bacteria® (MS)**					
The current study	171	32	46	18	3
The current study (adjusted)	171	-	79	-	21
Saudi Arabian adults with endodontic treatment [5]	100	27	25	26	22
Saudi Arabian adults without endodontic treatment [5]	100	38	32	11	19
**Dentocult SM®**					
Swedish children [3]	392	39	16	24	21
Swedish elderly people [4]	148	16	22	41	22
Hiyoshi Oral Health Clinics, 2015‡	3,109	13	16	34	37
Takamori Dental Practice, 2013§	1,478	9	23	36	32

†*n*: Number of participants. ‡Kumagai, T. personal communication, 10 April 2015. §Takamori, Y. personal communication, 25 May 2013.

‘Fluoride programme’

Relevant information on fluoride use was obtained through patient interviews. The interpretation of each score is as follows:

Score 0a: use of fluoridated water, fluoridated toothpaste and additional measure on a regular basis (a ‘maximum’ fluoride programme)

Score 0b: use of fluoridated water, fluoridated toothpaste and additional measure on an occasional basis (a ‘maximum’ fluoride programme)

Score 0c: use of fluoridated water and fluoridated toothpaste (a ‘maximum’ fluoride programme)

Score 0d: use of fluoridated toothpaste and additional fluoride on a regular basis (a 'maximum' fluoride programme)

Score 1: use of fluoridated water

Score 2: use of fluoridated toothpaste, *or*

Score 2: use of additional fluoride on a regular basis

Score 3: avoiding fluorides, not using fluoride toothpastes or other fluoride measures.

'Saliva secretion' parameter

The volume of stimulated saliva collected over five minutes was collected using CRT® saliva tests. Unstimulated saliva was not measured in the current study. In the dental practice with a normal appointment between 9 am and 5 pm, the medical-card patient sat upright and stimulated salivation by chewing a paraffin pellet for five minutes. The saliva was drooled into a disposable graduated test tube through a disposable funnel during the collection period. The dentist measured the volume of the saliva in the test tube from the lowest point on the meniscus, the measurement did not include the foam, if any. The four-level scoring system is as follows:

Score 0: ≥ 1.1 ml/minute (normal saliva secretion)

Score 1: < 1.1, ≥ 0.9 ml/minute (low stimulated saliva secretion)

Score 2: < 0.9, ≥ 0.5 ml/minute (low stimulated saliva secretion)

Score 3: < 0.5 ml/minute (very low, xerostomia).

'Buffer capacity'

CRT® buffer was used. Immediately after the stimulated was collected as described previously, the dentist used a disposable pipette to place some of this stimulated saliva on the test strip. After five minutes, the dentist compared the colour of the test strip with the standard colour chart. The scoring system for this parameter was performed as follows:

Score 0: High (normal or good buffering capacity)

Score 1: Medium (less than good buffering capacity)

Score 2: Low (low buffering capacity)

'Clinical judgement'

Just before risk assessment and randomisation were performed for the first group of the patients, it was revealed that the calculated average of '*chance of avoiding new cavities*' was higher than expected. Applying the increased risk score for the 'Clinical judgement' parameter, the average of '*chance of avoiding new cavities*' is similar to that of an Arabian study for an adult population with a similar mean age and mean DMFS [5]. Possible reasons were as follows:

1. Almost all patients used both fluoridated water and fluoridated toothpaste, which converted to the most favourable score for the 'Fluoride programme' parameter.
2. CRT® Bacteria (LB and MS) might be underscored.
3. The three-day food diary is self-reported and might lead to underscoring.
4. The reference data used for the 'Caries experience' parameter was from 15 years ago.
5. The eligibility criteria (medical-card-holder – proxy for low socioeconomic status – patients who have 20 or more than 20 teeth) may not adequately capture the lower socioeconomic group.

For adjusting such systematic situations, the use of the 'Clinical judgement' parameter is recommended (Hänsel Petersson, G. personal communication, 16 December 2011). The current study complied with this recommendation. Additional file Table 3 summaries the distribution and mean (SD) of '*chance of avoiding new cavities*' using both Score 1 (standard) and Score 2 (increased risk) for the 'Clinical judgement' parameter.

Additional file Table 3 Distribution (%) and mean (SD) of '*chance of avoiding new cavities*' with Scores 1 and 2 of 'Clinical judgement' (*n* = 171)

'*Chance of avoiding new cavities*'	'Clinical judgement'	
	With Score 1	With Score 2
0–20 (highest risk)	3.5	24.6
21–40	14.6	31.6
41–60	21.1	25.1
61–100 (lowest risk)	60.8	18.7
Mean (SD)	63.8 (21.0)	39.6 (21.9)
Min–Max	10–96	3–94

References

1. Whelton H, Crowley E, O'Mullane D, Woods N, McGrath C, Kelleher V, Guiney H, Byrtek M. Oral health of Irish adults 2000 - 2002. http://health.gov.ie/wp-content/uploads/2014/03/oral_health02.pdf. Accessed 10 November 2018.
2. Bratthall D, Hänsel Petersson G, Stjernswärd J. Cariogram Manual. http://www.mah.se/upload/FAKULTETER/OD/cariogram%20program%20caries/cariogmanual201net.pdf. Accessed 10 November 2018.
3. Hänsel Petersson G, Twetman S, Bratthall D. Evaluation of a computer program for caries risk assessment in schoolchildren. Caries Res. 2002;36:327-40.
4. Hänsel Petersson G, Fure S, Bratthall D. Evaluation of a computer-based caries risk assessment program in an elderly group of individuals. Acta Odontol Scand. 2003;61:164-71.

5. Merdad K, Sonbul H, Gholman M, Reit C, Birkhed D. Evaluation of the caries profile and caries risk in adults with endodontically treated teeth. Oral Surg Oral Med Oral Pathol Oral Radiol Endod. 2010;110:264-9.
